# Dry Eye Disease in Patients With Schizophrenia: A Case-Control Study

**DOI:** 10.3389/fmed.2022.831337

**Published:** 2022-02-09

**Authors:** Qiankun Chen, Zhengjiang Wei, Leying Wang, Xizhan Xu, Zhenyu Wei, Panpan Zheng, Kai Cao, Zijun Zhang, Kexin Chen, Qingfeng Liang

**Affiliations:** ^1^Beijing Institute of Ophthalmology, Beijing Tongren Eye Center, Beijing Tongren Hospital, Capital Medical University, Beijing Key Laboratory of Ophthalmology and Visual Sciences, Beijing, China; ^2^Beijing Miyun Mental Health Prevention and Treatment Hospital, Beijing, China; ^3^National Center for Children's Health, Beijing Children's Hospital, Capital Medical University, Beijing, China

**Keywords:** dry eye disease, schizophrenia, cytokine, inflammation, tear

## Abstract

**Objective:**

To evaluate the clinical features and inflammatory cytokines of dry eye disease (DED) in patients with schizophrenia.

**Methods:**

This is a case-control study. The modified self-rating depression scale (M-SDS) and the ocular surface disease index (OSDI) were used to evaluate the symptoms of depression and DED, respectively. Lipid layer thickness (LLT), partial blink rate (PBR), meibomian gland loss (MGL), tear break-up time (TBUT), corneal fluorescein staining, Schirmer I-test, and eyelid margin abnormalities were also measured. A multiplex ELISA Quantibody array was used to detect the inflammatory cytokines in the tears of all participants.

**Results:**

Forty schizophrenic patients and 20 control subjects were included. The mean age was 45.0 ± 9.5 years (range, 22–63 years) in schizophrenic patients and 45.4 ± 16.2 years (range, 23–76 years) in controls (*P* = 0.914). The ratio of male to female was 1.1 in schizophrenic patients and 1.0 in controls (*P* = 0.914). Ten women (52.6%) with schizophrenia and 2 (20%) in the control group (P = 0.096) were menopausal or post-menopausal. The OSDI [0.0 (0.0–4.2) vs. 7.3 (2.1–14.6)] and TBUT [4.5 (3.0–6.0) vs. 10.0 (3.5–11.0)] were significantly lower in patients with schizophrenia than in controls (*P* = 0.003 and *P* = 0.009, respectively). The rate of MGL [36.5 (17.5–47.5) vs. 8.5 (0.0–17.5)] increased in schizophrenic patients (*P* < 0.001). Among pro-inflammatory cytokines, the levels of interleukin (IL)-1α, IL-6, IL-11, IL-12A, IL-15, IL-17A, and granulocyte colony-stimulating factor (G-CSF) in tears were elevated in the schizophrenia group (all *P* < 0.01). Most of the chemokines examined were at increased levels in the tears of schizophrenics (all *P* < 0.05). The levels of matrix metalloproteinases-9 (MMP-9) and intercellular adhesion molecule-1 (ICAM-1) were also higher in the schizophrenic patients (all *P* < 0.001). The concentrations of IL-1Ra, tissue-inhibitor of metalloproteinase-1 (TIMP-1), and TIMP-2 in the schizophrenia group were decreased (all *P* < 0.001). In schizophrenic patients, the level of CCL2 in tears was positively correlated with OSDI (R = 0.34, *P* = 0.03). The increasing TIMP-1 and decreasing IL-5 were correlated with increasing LLT (*R* = 0.33, *P* = 0.035; *R* = −0.35, *P* = 0.027, respectively). The level of ICAM-1 was then positively correlated with partial blink rate (PBR) (*R* = 0.33, *P* = 0.035). There was a negative correlation between IL-8 and the Schirmer *I*-test (*R* = −0.41, *P* = 0.009).

**Conclusions:**

Patients with schizophrenia were more likely to experience asymptomatic DED, with mild symptoms and obvious signs. The inflammatory cytokines in the tears of schizophrenic patients differed greatly from that of non-schizophrenic patients.

## Introduction

Dry eye disease (DED) is one of the most common ocular surface diseases and has been recognized as a major public health problem (1). Based on the report of Dry Eye Workshop II (DEWS II) from the Tear Film & Ocular Surface Society, DED was defined as a multifactorial disease of the tear film and ocular surface, including three core parts: tear film instability, ocular surface inflammation, and neurosensory abnormalities (2). We have always emphasized that the symptoms were the main features of DED, but the symptoms of DED were often inconsistent with the signs (3). Some researchers, however, tried to explain it with the theory that mental health conditions could be one of the considerable contributing factors for DED symptoms and undiagnosed mental diseases could have similar symptoms in some special types of DED (4). In addition, psychiatric medications could influence the stability of the tear film and were considered as a risk factor for DED (5). In recent years, numerous studies have focused on the relationship between DED and psychiatric disorders, such as depression, anxiety, post-traumatic stress disorders, and sleep disorders (6–11).

Although some DED patients present psychiatric or mood disorders, it remains unclear whether psychiatric patients also experience the symptoms or clinical signs of DED. As a severe psychotic disorder, schizophrenia is characterized by varying degrees of cognitive impairments, emotional aberrations, and behavioral anomalies (12). So, It may be a good model to evaluate the relationship between DED and psychiatric disorders because schizophrenia always disrupts the processes of perception, reasoning, judgment. The medications for schizophrenia may also induce the signs of DED (13). Although a study conducted in Taiwan, China, found that patients with DED may be more likely to develop schizophrenia (10), the symptoms of DED were not associated with schizophrenia according to a population-based study in the Netherlands (14). Until now, there have been few studies to analyze the DED status of schizophrenic patients and detect the relationship between DED and schizophrenia. So, with the existing epidemiological investigations, the relationship between DED and schizophrenia was investigated at both the clinical and molecular levels.

## Materials and Methods

### Study Design

A case-control study was performed at Beijing Tongren Hospital, Capital Medical University, and Beijing Miyun Mental Health Prevention and Treatment Hospital between December 2020 and April 2021. This study was registered in the Chinese Clinical Trial Registry (Registration number: ChiCTR-RDD-17013855), and ethical approval was obtained from the Medical Ethics Committee of Beijing Tongren Hospital (TRECKY2019-130). All participants obtained written informed consent in accordance with the Declaration of Helsinki.

### Participants

All enrolled schizophrenic patients were hospitalized in the Beijing Miyun Mental Health Prevention and Treatment Hospital, whose diagnoses were conducted by professional psychiatrists (ZJ W) using international criteria for schizophrenia listed in the *Diagnostic and Statistical Manual of Mental Disorders* (Fourth Edition). The inclusion criteria were that the patients should be over 18 years old, their conditions should be under control, and they should be able to complete the questionnaires and ocular examinations. The exclusion criteria included current pregnancy or lactation, and any ocular surface disease that may affect the integrity of the cornea (for example, past corneal chemical injury, recurrent corneal epithelial erosion, corneal epithelial defect, and map-dot-fingerprint dystrophy). Cases with ocular topical treatment within the past 6 months, history of ocular trauma, previous ocular laser or surgical treatment, and other common ocular diseases were also excluded.

The inclusion criteria for the control group were that the patients should be older than 18 years with no ocular discomfort. Exclusion criteria of the control group included: (1) abnormality of lid margin and cornea under slit lamp microscopy; (2) conjunctival inflammation; (3) systemic diseases or medications that may cause DED; (4) contact lens wear in the month prior to enrollment; (5) other ocular diseases; (6) pregnant or lactating women.

### Demographic Information Collection

Demographic information was obtained from questionnaires based on the previous study (15). These questionnaires included basic information (age and gender), systemic medical history (such as hypertension, hyperlipidemia, diabetes mellitus, and coronary heart disease), and smoking and alcohol intake history.

### Depression Symptoms Evaluation

The symptoms of depression were assessed using the modified self-rating depression scale (M-SDS) adapted from the 1965 self-rating depression scale by Zung, which had been applied in the Beijing Eye Study (15, 16). The M-SDS consisted of 20 questions, each carrying a score of 1 to 4. Definite depression was defined as having an M-SDS score of 40 or higher.

### Ocular Surface Evaluation

The ocular surface symptoms of each participant were quantified by the ocular surface disease index (OSDI) questionnaire (range, 0–100) (17). OSDI contained 12 questions, which included DED symptoms (sensitivity to light, foreign body sensation eye pain or sore eyes, blurred vision and poor vision), restrictions on daily activities (reading, driving at night, working on the computer or automatic teller machine, and watching TV), and environmental factors (wind, low humidity, and air conditioning). Each question consisted of 5 levels, which were as follows: 0, none; 1, sometimes; 2, half the time; 3, most of the time; and 4, all the time. The higher the score, the more severe the symptoms. An OSDI score of 13 or higher was considered as a symptom of DED (18).

All ocular surface examinations were performed on the left eye of the participants.

The LipiView interferometer (TearScience Inc, Morrisville, North Carolina, USA) was used to measure lipid layer thickness (LLT), capture infrared photography of the meibomian gland, and analyze blink pattern as previously described (19). Interferometry color units (ICU) represent the amount of lipid distribution, and 1 ICU is about 1 nm of LLT. Conformance factor (CF) represents the quality of the measured data. If the CF is lower than 0.7, it must be re-tested to ensure the correctness of the data. Partial blink rate (PBR) was calculated by the ratio of partial blinks to total blinks (20). ImageJ software was used to semi-automatically analyze the loss rate of the lower lid meibomian glands.

Tear break-up time (TBUT) was calculated according to the previous description (21). In brief, the fluorescein strip (Tianjin Jingming New Technological Development Co., Ltd., China) was wetted with saline, and then a drop was put into the lower fornix. Participants were asked to blink several times before being observed under a cobalt blue filter at the slit lamp. The time gap between participants starting to open their eyes and the appearance of the first dark spot on the tear film was recorded. The average of three consecutive measurements was regarded as TBUT. TBUT <10 s was considered abnormal (18).

Corneal staining was conducted according to the Oxford scheme to assess the integrity of corneal epithelium after TBUT evaluation (22). In brief, the Oxford score was graduated with points ranging from 0 to 5, based on the staining dots of cornea as follows: 0 for ≤ 1 dot; 1 for ≤ 10 dots; 2 for ≤ 32 dots; 3 for ≤ 100 dots; 4 for ≤ 316 dots; and 5 for >316 dots.

Schirmer *I*-test was carried out as described previously (23). A 5 × 35 mm sterile filter paper strip (Tianjin Jingming New Technological Development Co., Ltd., China) with one end folded at a right angle at 5 mm was placed at the middle and outer 1/3 of the inferior fornix. The subject was then asked to close the eyes, and the soaked length of the filter paper was measured after 5 min.

Eyelid margin abnormalities (including irregularity, telangiectasia, plugging of orifices, and Marx line location) were evaluated under slit-lamp microscopy with 1 point for each item, and the total score of eyelid margin was added up (24).

### Tear Collection and Cytokine Detection

A total quantity of 60 μl of tears were extracted from the temporal area of the meniscus under the left eye of each participant using 20 μl microcaps (Drummond Scientific Company, Broomall, PA, USA) and stored at −80°C.

Multiplex ELISA Quantibody array kits (QAH-DED-1, RayBiotech) were used to detect the cytokines in tears, including interleukins (ILs), chemokines, matrix metalloproteinases (MMPs) and inhibitors, and other cytokines related to DED. The Quantibody array was carried out according to the manual provided by the company. To be brief, the 16-well glass chip was dried completely and incubated at room temperature for 1 h with 100 μl sample dilution in each well. The diluent was removed and cleaned two times by Wellwash Versa (Thermo Fisher Scientific, Waltham, MA, USA) with wash buffer I and II, respectively. Samples of 60 μl volume were added and incubated overnight at 4°C. Detection antibody cocktails of 80 μl were then added and incubated at room temperature for 2 h on a shaker. After cleaning again as before, 80 μl of Cy3 equivalent dye-conjugated streptavidin was put into each well and incubated on a shaker at room temperature, away from light, for 1 h. After the third cleaning, a laser scanner (InnoScan 300 Microarray Scanner, Innopsys, Carbonne, France) was used for detection. Finally, QAH-DED-1 data analysis software was used for data analysis. Standard substances with eight concentration gradients were used as controls.

### Statistical Analysis

Statistical analysis was performed with SPSS 26.0. A Kolmogorov-Smirnov test was used to assess for normality of continuous variables. The *t*-test was used for measurement data that followed a normal distribution, described by mean ± standard deviation. For non-normal distribution parameters, the Mann–Whitney *U*-test was used, and the values of parameters were described in terms of median and inter-quartile range. Chi-square test was used for enumeration date. Moreover, Spearman's correlation coefficient was calculated. The value of *P* < 0.05 was considered statistically significant.

## Results

### Demographic Characteristics

A total of 60 participants were included−40 patients with schizophrenia and 20 control subjects. The demographic information and medical history of the schizophrenic patients and controls were summarized in Table 1. The mean age was 45.0 ± 9.5 years (range, 22–63 years) in the schizophrenic group and 45.4 ± 16.2 years (range, 23–76 years) in the control group (*P* = 0.914). The number of men in the schizophrenic group and control group were 21 (52.5%) and 10 (50%), respectively (*P* = 0.802). Ten women (52.6%) with schizophrenia and two (20%) in the control group (*P* = 0.096) were menopausal or post-menopausal. The length of hospital stay in patients with schizophrenia was 33.4 ± 65.2 months (range, 1–315 months). Twenty-six (65%) patients took one kind of antipsychotic drug and 14 (35%) patients took a combination of antipsychotic drugs. These antipsychotic drugs included rispehdone, olanzapine, zopiclone, clonazepam, sodium valproate, trihexyphenidyl, lorazepam, haloperidol, quetiapine fumarate, magnesium valproate, sertraline, escitalopram oxalate, and estazolam.

**Table 1:** Characteristics and basic medical history of schizophrenic patients and normal controls.

**Parameters**	**Schizophrenia group (*n* = 40)**	**Normal controls (*n* = 20)**	***P*-value**
Age (years)	45.0 ± 9.5	45.4 ± 16.2	0.914
**Gender [*****n*** **(%)]**			0.802
Male	19 (47.5%)	10 (50%)	
Female	21 (52.5%)	10 (50%)	
**Systemic diseases [*****n*** **(%)]**			
Hypertension	6 (15%)	1 (5%)	0.407
Hyperlipidemia	8 (20%)	1 (5%)	0.249
Diabetes mellitus	5 (12.5%)	2 (10%)	1.000
Coronary heart disease	3 (7.5%)	1 (5%)	1.000
Others	10 (25%)	6 (30%)	0.542
Smoking [*n* (%)]	19 (47.5%)	3 (15%)	0.022^*^
Alcohol intake [*n* (%)]	4 (10%)	2 (10%)	0.089
M-SDS	26.0 (22.0–32.5)	26.5 (23.0–30.5)	0.597

* *P < 0.05 was considered statistically significant*.

There was no significant difference in the prevalence of systemic diseases (such as hypertension, hyperlipidemia, diabetes mellitus, and coronary heart disease) (all *P* < 0.05). Nineteen (47.5%) patients with schizophrenia smoked more than 5 cigarettes a day, far more than the 3 (15%) patients in the controls (*P* = 0.022). Four (10%) schizophrenic patients and 2 (10%) of the control subjects consumed alcohol everyday (*P* = 0.089). The median score of M-SDS in the schizophrenia group was 26.0 (22.0–32.5), which was similar to 26.5 (23.0–30.5) of the controls (*P* = 0.597).

### Clinical Manifestations

Compared with normal controls, schizophrenic patients had lower OSDI score [median 0.0 (0.0–4.2) vs. 7.3 (2.1–7.6); *P* = 0.003, Table 2]. Three (7.5%) patients with schizophrenia showed DED symptoms, which was <5 (25%) patients in the controls (*P* = 0.073). However, the rate of meibomian gland loss (MGL) was 36.5 (17.5–47.5) percent in schizophrenic patients compared with 8.5 (0.0–17.5) percent in controls (*P* < 0.001). In the assessment of tear film stability, TBUT was significantly reduced in schizophrenia group (*P* = 0.009). Notably, 39 (97.5%) of schizophrenics and 9 (45%) of controls presented abnormalities in TBUT (*P* < 0.001). There was no significant difference between the two groups in LLT, PBR, Oxford score, and the Schirmer *I*-test (all *P* > 0.05). Therefore, among schizophrenic patients, 36 (90%) cases presented DED signs without symptoms, which were more than 5 (25%) from the control group (*P* < 0.001). In evaluation of eyelid margin, 14 (35.0%) schizophrenic patients and 1 (5.0%) in the control group (*P* = 0.009) presented irregularity. There was no significant difference in telangiectasia, plugging of orifices, and Marx line location between the two groups (*P* = 0.204; *P* = 0.102; *P* = 0.463, respectively). The total score of eyelid margin was higher in schizophrenic patients than controls (median, 2.0 vs. 1.5; *P* = 0.032).

**Table 2:** Ocular surface clinical outcomes in controls and schizophrenia group.

**Parameters**	**Schizophrenia group (*n* = 40)**	**Normal controls (*n* = 20)**	***P*-value**
OSDI	0.0 (0.0–4.2)	7.3 (2.1–14.6)	0.003^*^
LLT (nm)	75 (58.5–84)	89.5 (63.0–100.0)	0.229
PBR (%)	50.0 (25.0–77.5)	77.4 (33.3–100.0)	0.229
MGL (%)	36.5 (17.5–47.5)	8.5 (0.0–17.5)	<0.001^*^
TBUT (s)	4.5 (3.0–6.0)	10.0 (3.5–11.0)	0.009^*^
Oxford score	0.0 (0.0–0.0)	0.0 (0.0–0.0)	0.294
Schirmer *I*-test (mm)	10.0 (6.0–16.0)	7.0 (2.5–17.5)	0.509
**Eyelid margin** **[*****n*** **(%)]**			
Irregularity	14 (35.0)	1 (5.0)	0.009^*^
Telangiectasia	24 (60.0)	9 (45.0)	0.204
Orifice plugging	30 (75.0)	11 (55.0)	0.102
Marx line location	22 (55.0)	10 (50.0)	0.463
Score of eyelid	2.0 (1.0–3.0)	1.5 (1.0–2.0)	0.032^*^

*OSDI, ocular surface disease index; LLT, lipid layer thickness; PBR, partial blink rate; TBUT, tear brake-up time; MGL, meibomian gland loss*.

* *P < 0.05 was considered statistically significant*.

### Cytokine Expression in Tears

**Figure 1:**
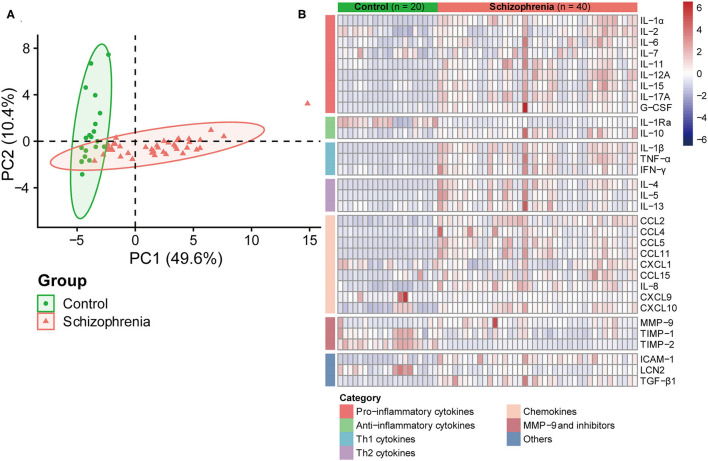
Cytokines profile in tears of subjects with and without schizophrenia. **(A)** Principal component analysis was performed by the concentration of 32 cytokines in tears. Scatter plot of principal component 1 (PC1) and PC2 were represented, and each dot represented a sample projected in them. The orange and green dots represented schizophrenia and control groups, respectively. **(B)** Heatmap of high and low concentrations of tear cytokines. The groups evaluated were controls (*n* = 20) and patients with schizophrenia (*n* = 40). High levels of cytokines were shown in red on the heatmap, while low levels were shown in blue.

The expression levels of cytokines in tears were measured between the two groups and clustered into several categories according to previous studies (25, 26). Principal component analysis (PCA) and the heatmap showed obvious separation of cytokines from the two cohorts (Figure 1), suggesting that the ocular surface status of the two populations may be different.

**Figure 2:**
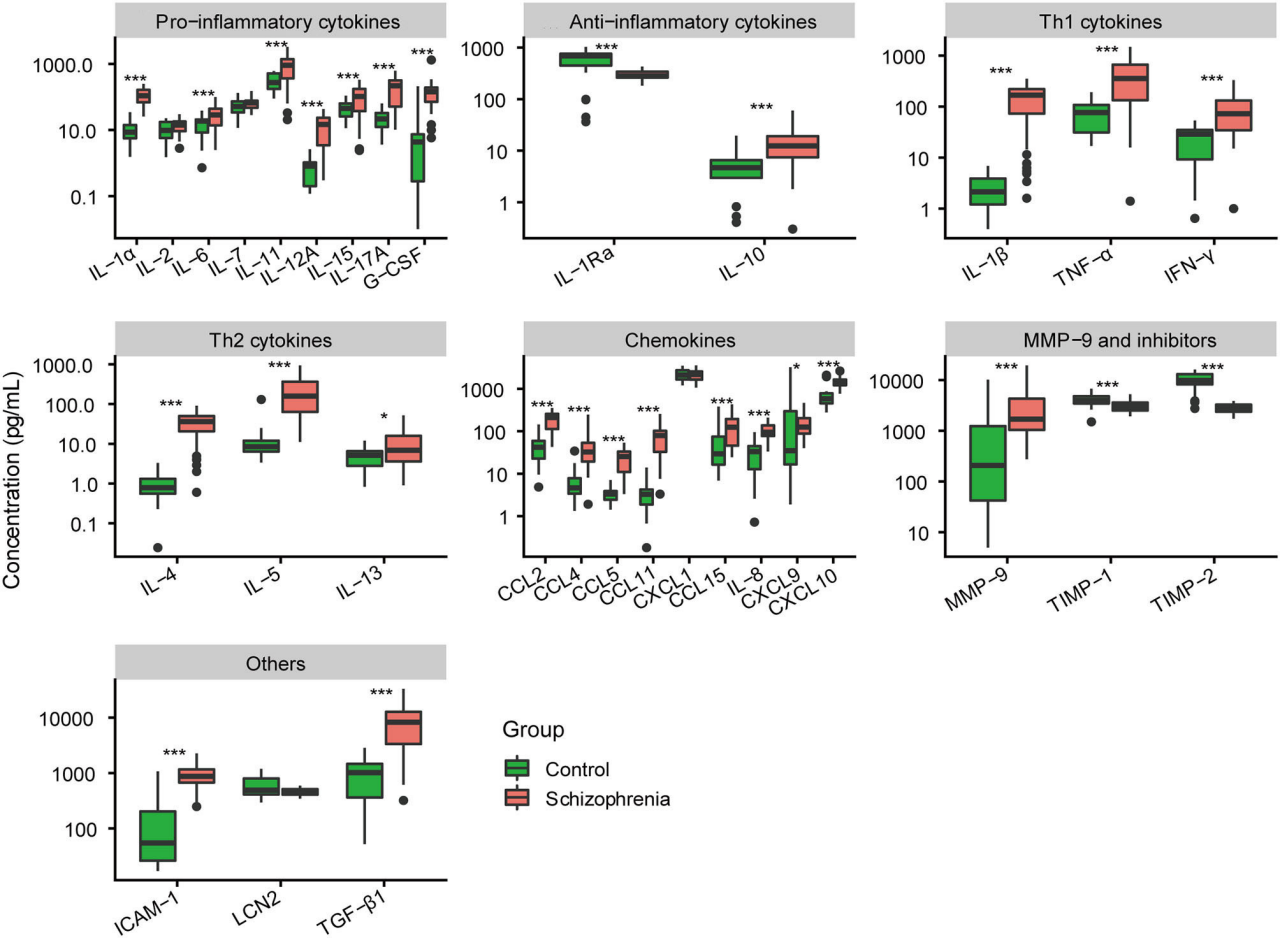
Comparative analysis of expression level of 32 cytokines in tears between normal controls and schizophrenia group. *P < 0.05; ***P < 0.001.

Among pro-inflammatory cytokines, the levels of interleukin (IL)-1α, IL-6, IL-11, IL-12A, IL-15, IL-17A, and granulocyte colony-stimulating factor (G-CSF) in tears were significantly higher in patients with schizophrenia than that in normal controls (all *P* < 0.01; Figure 2). No significant differences in IL-2 and IL-7 levels were observed between the two groups (*P* = 0.107; *P* = 0.163, respectively). Among anti-inflammatory cytokines, IL-1Ra was decreased and IL-10 was increased in tears of schizophrenic patients compared with that of controls (all *P* < 0.001).

The cytokines of tears released by T helper (Th) cells were also measured. The cytokines secreted by Th1 were significantly higher in the tears of schizophrenic patients than that of normal controls, including IL-1β, tumor necrosis factor (TNF)-α, and interferon (IFN)-γ (all *P* < 0.001; Figure 2). The cytokines produced by Th2 (IL-4, IL-5, and IL-13) were also increased in patients with schizophrenia (*P* < 0.001; *P* < 0.001; *P* = 0.012, respectively).

In chemokines, the expression levels of chemokine (C-C motif) ligand 2 (CCL2), CCL4, CCL5, CCL11, CCL15, IL-8, chemokine (C-X-C motif) ligand 9 (CXCL9), and CXCL10 in tears of schizophrenic patients were significantly higher than that of normal controls (all *P* < 0.05; Figure 2). There was no significant difference in CXCL1 level of tears between schizophrenic patients and controls (*P* = 0.589).

Additionally, we also found that the expression of matrix metalloproteinases-9 (MMP-9) and their inhibitors in tears were different between the two groups. The level of MMP-9 was increased in schizophrenic patients (*P* < 0.001; Figure 2). However, the concentration of tissue-inhibitor of metalloproteinase-1(TIMP-1) and TIMP-2 in patients was much lower than that of controls (all *P* < 0.001). Finally, other cytokines outside the above classification, including intercellular adhesion molecule-1 (ICAM-1) and transforming growth factor-beta 1 (TGF-β1), were also elevated in the tears of schizophrenic patients (all *P* < 0.001).

### Correlation Between Clinical Parameters and Cytokines

**Figure 3:**
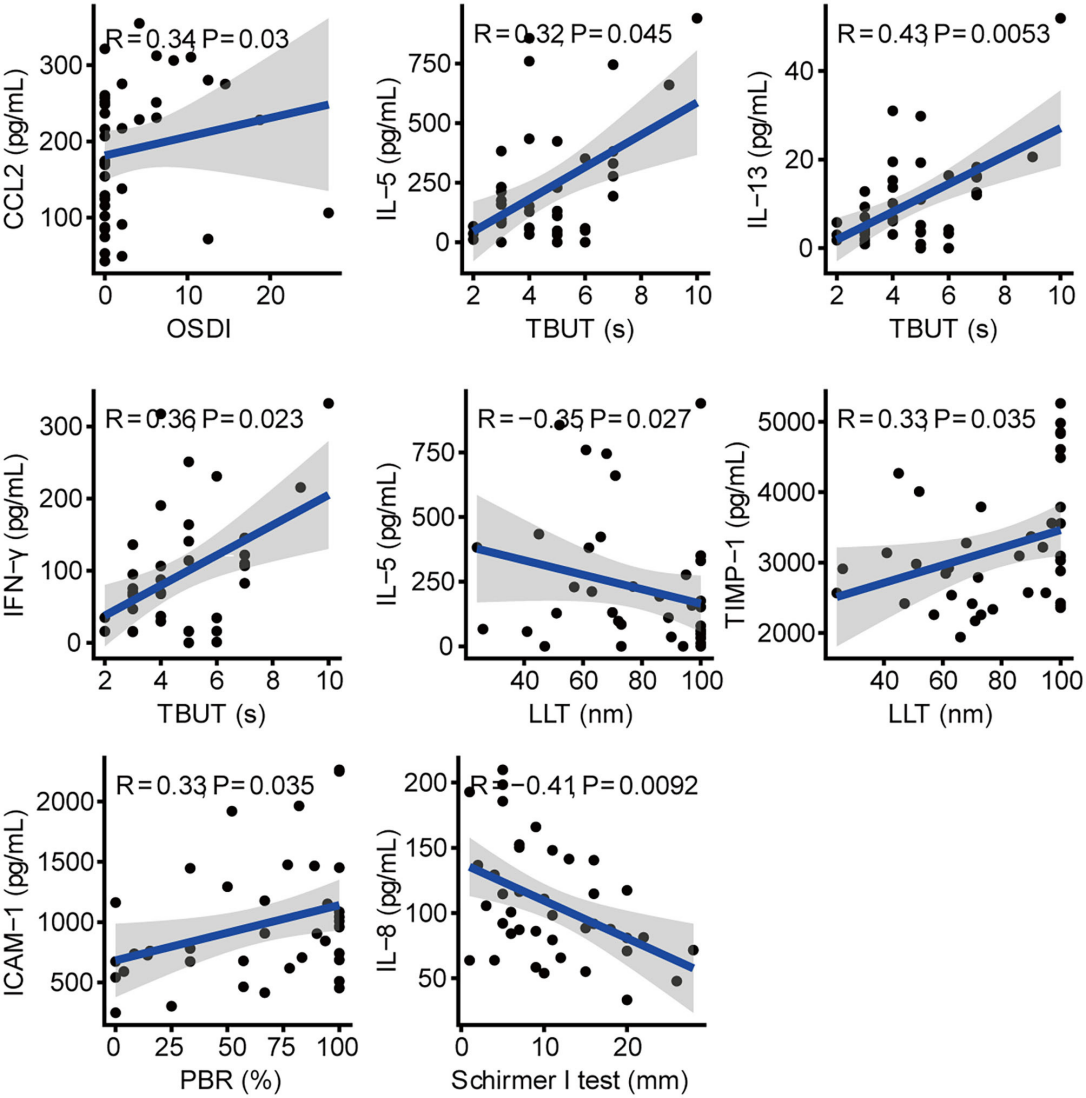
Correlation between cytokines and clinical parameters in schizophrenic patients.

Among schizophrenic patients, the concentration of CCL2 in tears was positively correlated with OSDI (*R* = 0.34, *P* = 0.03; Figure 3). The levels of IL-13, IFN-γ, and IL-5 were positively correlated with TBUT (*R* = 0.43, *P* = 0.005; *R* = 0.36, *P* = 0.023; *R* = 0.32, *P* = 0.045, respectively). The increasing TIMP-1 and decreasing IL-5 were correlated with increasing LLT in schizophrenic patients (*R* = 0.33, *P* = 0.035; *R* = −0.35, *P* = 0.027, respectively). The increasing level of ICAM-1 was positively correlated with increasing PBR in patients with schizophrenia (*R* = 0.33, *P* = 0.035). There was a negative correlation between IL-8 and Schirmer *I*-test (*R* = −0.41, *P* = 0.009). In addition, we also found the correlation of cytokines in the tears of patients with schizophrenia (Supplementary Figure S1).

## Discussion

In the current study, we found an increase in DED (mainly asymptomatic DED) in patients with schizophrenia. Compared to controls, most inflammatory cytokines of tears were significantly increased in schizophrenic patients. Correlation between the concentration of cytokines and clinical parameters was also observed.

The results of this study showed that OSDI and TBUT were decreased, and the rate of MGL was elevated in patients with schizophrenia. These indicated that the symptoms and signs of DED in schizophrenic patients were inconsistent, with mild symptoms and severe signs. Moreover, low TBUT, high MGL, and normal Schirmer *I*-test suggested that DED in schizophrenic patients was more likely to be evaporative rather than aqueous-deficient. A study conducted by Tiskaoglu et al. (27) showed that patients with newly diagnosed depressive disorder had lower Schirmer score and TBUT, while DED symptoms were not different from the control group. Kim et al. (28) suggested that depression was related to DED symptoms rather than signs. A review by McMonnies (29) also showed that dry eye features in patients with mental disorders were mostly severe symptoms and relatively mild signs. In addition, other studies have found that patients with depression, anxiety, and obsessive-compulsive disorder had more severe signs and symptoms of DED than the controls (11, 30). Unlike previous studies, our study involved patients with schizophrenia. Moreover, the patients in the previous studies were outpatients, whereas we focused on inpatients. In the current study, all schizophrenic patients took antipsychotic drugs, which could induce side-effects on the ocular surface, including pigmentary deposits of conjunctiva and cornea, reduction of tear secretion and tear film stability, damage of corneal epithelium, reduction of corneal thickness, and ocular toxicity (13, 31, 32). The absence of DED symptoms in patients with schizophrenia may be due to cognitive abnormalities and hypoesthesia (33). Moreover, the obvious signs of DED in schizophrenic patients could be due to the lack of activities and use of antipsychotic drugs (13).

Cytokines are key signal molecules of the immune system, which play important roles in the inflammatory response of the ocular surface and whole body. Among the tear cytokines examined, increased levels of pro-inflammatory cytokines (IL-1α, IL-6, IL-11, IL-12A, IL-15, IL-17A, and G-CSF), anti-inflammatory cytokines (IL-10), Th1 cytokines (IL-1β, TNF-α, and IFN-γ), Th2 cytokines (IL-4, IL-5, and IL-13), chemokines (CCL2, CCL4, CCL5, CCL11, CCL15, IL-8, CXCL9, and CXCL10), MMP-9, and others (ICAM-1 and TGF-β1) were observed in patients with schizophrenia. While the concentration of IL-1Ra of anti-inflammatory cytokines, TIMP-1 and TIMP-2 were decreased in tears of schizophrenic patients. A meta-analysis showed increased levels of IL-6, IL-8, IL-10, IL-12, IL-1β, IFN-γ, TNF-α, and TGF-β in the blood of patients with schizophrenia, which was similar to our findings (34). The levels of IL-2, IL-4, IL-10, TGF-β, and IL-17 in blood were also detected to be elevated in schizophrenic patients (35). Some studies found an increased level of IL-1Ra in the serum of patients with schizophrenia, while another study showed decreased expression of IL-1Ra in the prefrontal cortex (34–36). In chemokines, the levels of CCL2, CCL3, CCL4, CCL5, CCL11, and IL-8 in serum were increased in patients compared to controls, but CXCL9 and CXCL10 were similar between the two groups (37, 38). MMP-9, as a novel role involved in pathological synaptic plasticity in schizophrenia, was highly expressed in serum and brain tissues of patients with schizophrenia and could be regulated by TIMP-1 and TIMP-2 (39). The ratio of MMP-9/TIMP-1 in serum was higher in schizophrenic patients compared to healthy controls, which was similar to our study (40). As inflammation of the ocular surface is one of the core mechanisms of DED, increased inflammatory cytokines in the tears of schizophrenic patients could potentially cause ocular surface damage and promote the occurrence of DED. In a meta-analysis of 13 studies, there were strong pieces of evidence that the concentrations of IL-1β, IL-6, IL-10, IFN-γ, and TNF-α were significantly elevated in the tears of DED patients (41). The levels of IL-1α, IL-4, IL-11, IL-12A, IL-13, IL-15, IL-17A, and G-CSF were also increased in the tears of DED patients according to previous studies (42–45). Additionally, IL-1Ra was reduced in patients with DED, and topical treatment with IL-1Ra was effective in improving the clinical signs and reducing underlying inflammation of DED (46). During the inflammation response of DED, chemokines such as CCL2, CCL3, CCL5, CCL15, IL-8, CXCL9, and CXCL10 could recruit and bind macrophages, dendritic cells, neutrophils, and activated T cells (47–49). MMP-9 was a recognized cytokine that activated other inflammatory cytokines, leading to a vicious cycle of inflammation, impaired tear secretion, ocular surface damage, and worsening DED symptoms (50). Matrix metalloproteinase inhibitors, TIMP-1 and TIMP-2, could mediate corneal regeneration by inducing corneal epithelium proliferation and inhibiting apoptosis (51). Another study showed that the ratio of MMP-9/TIMPs was higher in evaporative dry eye, which was consistent with the current study (52). ICAM-1 and TGF-β1 were also highly expressed in the ocular surface of patients with DED (53, 54).

Further study found the correlation between tear cytokine levels and DED clinical parameters. As a well-known monocyte chemotactic protein-1 (MCP-1), the increase in CCL2 concentration was positively correlated with the increase of OSDI score in this study, suggesting that CCL2 could be an aggravating factor for the symptoms of DED. Similar to our findings, Perez et al. (55) found a significant increase of CCL2 in DED patients with mild signs but exaggerated symptoms. IL-8, a chemokine that recruited immune cells, was increased in the tears of patients with DED and has been found to be negatively associated with the Schirmer *I*-test, the same as in our study (56). As a natural endogenous inhibitor of MMP-9, TIMP-1 improved the symptoms and signs of DED, which was consistent with the positive correlation between the increase in TIMP-1 and the increase in LLT in the current study (57). IL-13 and IFN-γ were associated with the damage of corneal epithelial integrity and decrease of tear secretion in evaporative dry eye (58, 59). However, the levels of IL-13 and IFN-γ in tears were positively correlated with TBUT, as observed by us. Increased concentration of IL-5 in tears was inversely associated with TBUT, OSDI, and Oxford score in patients with DED, whereas we observed a positive association between IL-5 and TBUT, and a negative correlation between IL-5 and LLT in patients with schizophrenia (59, 60). In addition, we also observed that ICAM-1 concentration was positively correlated with PBR in patients. ICAM-1, an adhesion molecule that recruited and assisted in the migration of inflammatory cells during inflammation response of DED, has been a target for dry eye therapy (55). Also, partial blink was reported to be associated with DED (61). The correlation between cytokines and clinical parameters was somewhat different from previous studies, possibly because the ocular surface conditions of patients with schizophrenia may be different from those of patients with ordinary DED.

There were some limitations in this study. First, the relationship of inflammatory cytokines between blood and tears was not comparatively analyzed because of ethical issues. Second, all patients with schizophrenia were treated with antipsychotic medicines, which may have a potential effect on the results. Third, more patients with schizophrenia smoked than controls, which was consistent with the results of a meta-analysis that smoking may be associated with the risk of dry eye (62). Finally, a small number of cases were included in this study. There is a requirement for a prospective study with a large sample size to verify our findings.

In conclusion, patients with schizophrenia were more likely to be accompanied by asymptomatic DED, which presented mild symptoms and obvious signs. DED in patients with schizophrenia was a mixed type, but mainly evaporative. The cytokine profiles in tears of schizophrenic patients differed from that of non-schizophrenic patients. Correlations between tear cytokines and ocular surface clinical parameters were also observed. Further studies of DED in untreated schizophrenic patients are needed.

## Data Availability Statement

The original contributions presented in the study are included in the article/Supplementary Material, further inquiries can be directed to the corresponding author/s.

## Ethics Statement

The studies involving human participants were reviewed and approved by Medical Ethics Committee of Beijing Tongren Hospital (TRECKY2019-130). The patients/participants provided their written informed consent to participate in this study.

## Author Contributions

QL performed the study design. QC analyzed the data and wrote the manuscript. QC, ZJW, LW, XX, ZW, PZ, KC, ZZ, and KXC collected and analyzed data. All authors approved the final manuscript.

## Funding

This work was supported by the National Natural Science Foundation of China grants to QL (No. 81970765).

## Conflict of Interest

The authors declare that the research was conducted in the absence of any commercial or financial relationships that could be construed as a potential conflict of interest.

## Publisher's Note

All claims expressed in this article are solely those of the authors and do not necessarily represent those of their affiliated organizations, or those of the publisher, the editors and the reviewers. Any product that may be evaluated in this article, or claim that may be made by its manufacturer, is not guaranteed or endorsed by the publisher.
