# Supplementary material for: Dry Eye Disease in Patients With Schizophrenia: A Case-Control Study
Source: Front Med (Lausanne). 2022 Feb 9;9:831337. doi: 10.3389/fmed.2022.831337 (PMC8864171; doi:10.3389/fmed.2022.831337)
Supplement: Supplementary file 1 [file Data_Sheet_1.docx]

**Figure S1.** Correlation of tear cytokines in patients with schizophrenia. The pink squares indicated a significantly positive correlation. The darker the color, the higher the correlation.
