# Supplementary figures and images for: Dry Eye Disease in Patients With Schizophrenia: A Case-Control Study
Source: Front Med (Lausanne). 2022 Feb 9;9:831337. doi: 10.3389/fmed.2022.831337 (PMC8864171; doi:10.3389/fmed.2022.831337)

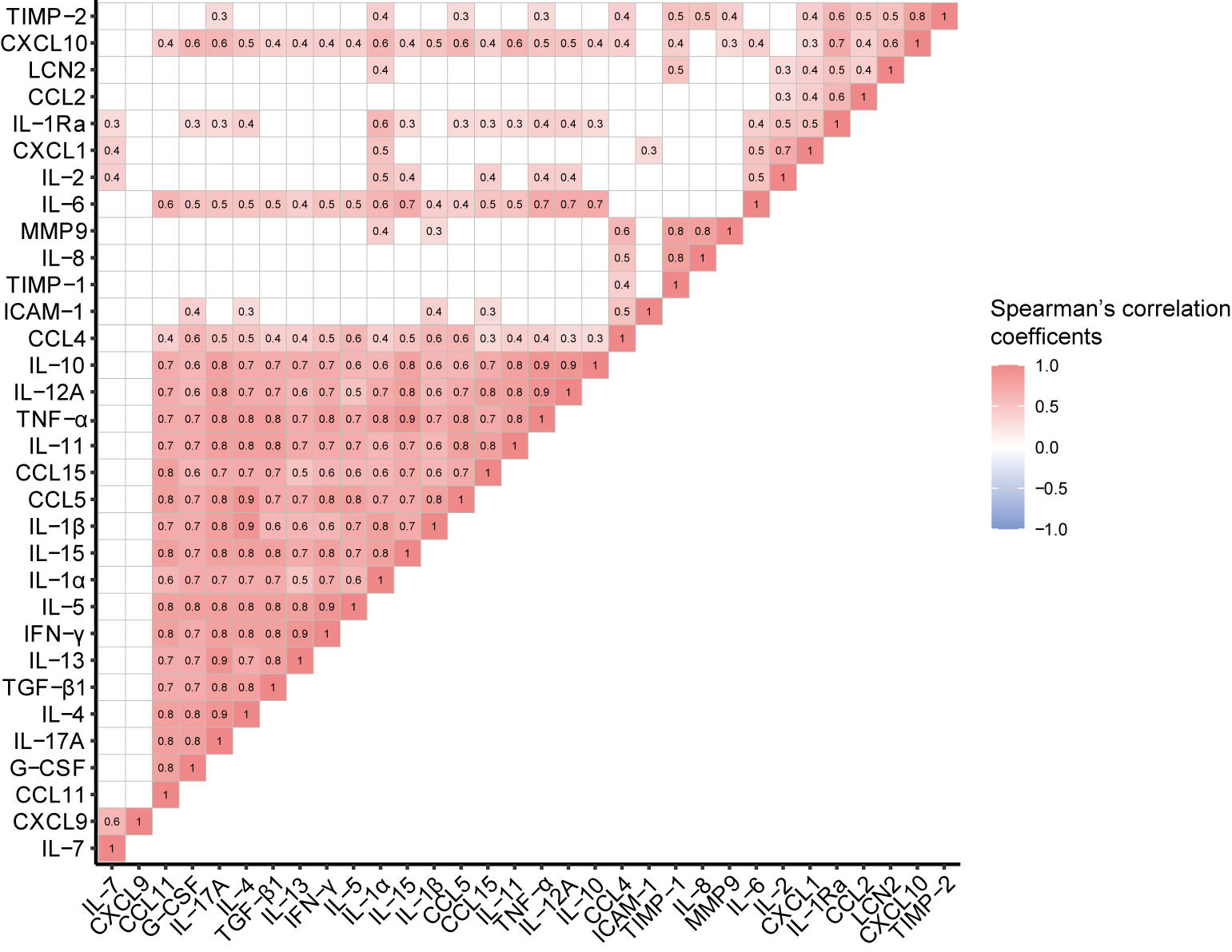

Supplement: Supplementary file 2 [file Image_1.TIF]
